# CCN5/WISP-2 restores ER-∝ in normal and neoplastic breast cells and sensitizes triple negative breast cancer cells to tamoxifen

**DOI:** 10.1038/oncsis.2017.43

**Published:** 2017-05-22

**Authors:** S Sarkar, A Ghosh, S Banerjee, G Maity, A Das, M A Larson, V Gupta, I Haque, O Tawfik, S K Banerjee

**Affiliations:** 1 Cancer Research Unit, Kansas City VA Medical Center, Kansas City, MO, USA; 2 Department of Anatomy and Cell Biology, University of Kansas Medical Center, Kansas City, KS, USA; 3 Division of Hematology and Oncology, Department of Medicine, University of Kansas Medical Centre, Kansas City, KS, USA; 4 Department of Pathology and Laboratory Medicine, University of Kansas Medical Center, Kansas City, KS, USA; 5 Transgenic and Gene-targeting Institutional Facilities, University of Kansas Medical Centre, Kansas City, KS, USA

## Abstract

CCN5/WISP-2 is an anti-invasive molecule and prevents breast cancer (BC) progression. However, it is not well understood how CCN5 prevents invasive phenotypes of BC cells. CCN5 protein expression is detected in estrogen receptor-α (ER-α) -positive normal breast epithelial cells as well as BC cells, which are weakly invasive and rarely metastasize depending on the functional status of ER-α. A unique molecular relation between CCN5 and ER-α has been established as the components of the same signaling pathway that coordinate some essential signals associated with the proliferation as well as delaying the disease progression from a non-invasive to invasive phenotypes. Given the importance of this connection, we determined the role of CCN5 in regulation of ER-α in different cellular settings and their functional relationship. In a genetically engineered mouse model, induced expression of CCN5 in the mammary ductal epithelial cells by doxycycline promotes ER-α expression. Similarly, CCN5 regulates ER-α expression and activity in normal and neoplastic breast cells, as documented in various *in vitro* settings such as mouse mammary gland culture, human mammary epithelial cell and different BC cell cultures in the presence or absence of human recombinant CCN5 (hrCCN5) protein. Mechanistically, at least in the BC cells, CCN5 is sufficient to induce ER-α expression at the transcription level via interacting with integrins-α6β1 and suppressing Akt followed by activation of FOXO3a. Moreover, *in vitro* and *in vivo* functional assays indicate that CCN5 treatment promotes response to tamoxifen in triple-negative BC (TNBC) cells possibly via restoring ER-α. Collectively, these studies implicates that the combination treatments of CCN5 (via activation of CCN5 or hrCCN5 treatment) and tamoxifen as potential therapies for TNBC.

## Introduction

Estrogen receptor-α (ER-α), a ligand-dependent transcription factor,^1^ has an important role in sexual development, reproductive functions, neuroendocrine functions, cardiovascular functions and carcinogenesis in breast.^2, 3, 4, 5^ Although a subset of non-proliferating epithelial cells express ER-α in rodent and human mammary glands,^6, 7^ ER-α is indispensable for the growth and morphogenesis of the adult mammary gland.^8^ Consequently, studies suggested that the ER-α-mediated activation of paracrine signaling pathways^9, 10^ may promote proliferation of neighboring ER-α-negative epithelial cells and morphogenesis in mammary gland.^8^

Unlike most of the normal mammary epithelial cells, the majority (~75%) of human breast cancers (BC) and precursor lesions express high levels of ER-α.^11^ Moreover, higher ER-α expression was found in the mammary epithelial cells of female populations who are at higher risk for BC compared to the populations at relatively low risk for BC incidence.^12^ Interestingly, deregulation, dysfunction or suppression of ER-α has been found to involve in tumor aggressiveness, metastasis and possibly hormone resistance.^13, 14^ In the transgenic mouse model, ER-α overexpression in mammary epithelial cells is associated with the precursor lesions^15^ and tumor growth with no aggressive phenotypes.^16, 17, 18, 19^ Although ER-α has emerged as an important factor for physiological and pathophysiological events in breast over the past decade, the mechanisms of regulation of ER-α in the breast epithelial cells are still unknown. Previously, two studies suggested that ER-α expression can be regulated in BC cells by p53^20^ and Twist.^21^ However, p53 or Twist do not regulate ER-α in normal mammary epithelial cells while being constitutively expressed in these cells^22, 23^ or overexpressed by inducers in BC cells (Banerjee, unpublished). Thus, it is still unclear what micro-environmental scenario decides ER-α status in normal breast epithelial cell or malignant cells for aforesaid diverse functions.

CCN5 (previously known as WISP-2), a matricellular protein, is expressed in normal and non-invasive breast epithelial cells and is becoming an increasingly important focus in BC research.^24, 25, 26^ Multiple studies have shown that CCN5-overexpressed BC cells are less aggressive in nature compared to CCN5-under-expressed or -negative BC cells. Moreover, CCN5 expressing BC cells are always ER-α positive, while CCN5 expression is lacking in HER-2/Neu positive and triple-negative BC (TNBC) cells.^25, 27, 28, 29, 30, 31^ Ectopic CCN5 expression augments ER-α expression in ER-α-negative BC cells.^25, 32^ Collectively, these studies implicate a fine tune between CCN5 signaling and ER-α pathways in BCs. However, the mechanism of CCN5 regulation of ER-α and functional significance have not yet been fully elucidated. This study aims to gain a better understanding of the relationship between CCN5 and ER-α in normal and cancer cells, the molecular basis of restoring ER-α by CCN5 in TNBC cells, and finally, the efficacy of tamoxifen (Tam) in TNBC cells by combination treatment of Tam and human recombinant CCN5 (hrCCN5) protein using rational *in vitro* and *in vivo* models.

## Results

### CCN5 augments ER-α expression in normal human mammary epithelial cells and ductal epithelial cells of mouse mammary glands under *in vitro* conditions

**Figure 1:**
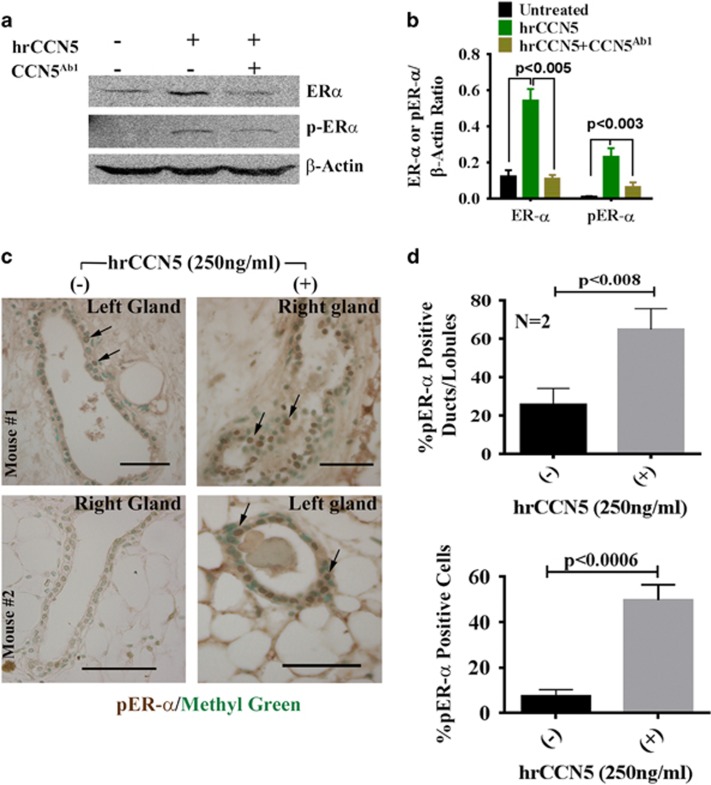
CCN5 regulates constitutive and active forms of ER-α in normal HMECs and ductal epithelial cells of mouse mammary gland. (**a** and **b**) Representative western blot (**a**) and quantification (**b**) of ER-α and p-ER-α (active form) protein level in HMECs treated with or without human recombinant protein (hrCCN5 (250 ng/ml)) or in combination of hrCCN5 and CCN5 antibody (500 ng/ml) for 48 h. All data represent means ±s.e.m. of three independent experiments. *P-*values were calculated using one way analysis of variance and two-tailed unpaired Student’s *t*-test. (**c** and **d**): Immuno-histochemical localization (**c**) and quantification (**d**) of pER-α in the ducts and lobules of mouse mammary glands cultured as indicated for 7 days in the presence or absence of hrCCN5 (250 ng/ml). Arrows indicate the ER-α positive ductal cell in glands of different FVB/N mice. Scale bars, 100 μm. Data are presented as mean±s.e.m. (*n*=2 mice). *P*-values were calculated using two-tailed unpaired Student’s *t*-test.

On the basis of a previous hypothesis that CCN5 could be a regulator of ER-α,^25, 33^ we sought to determine whether CCN5 is sufficient to upregulate the expression and activity of ER-α in mammary ductal epithelial cells. To do so, we examined the effect of hrCCN5; (250 ng/ml for 48 h) on the expression of ER-α and pER-α in normal human mammary epithelial cells (HMECs) in the presence or absence of a CCN5 neutralizing antibody (500 ng/ml). We found that both ER-α and pER-α protein levels were significantly increased in hrCCN5-treated HMECs compared to untreated cells (Figures 1a and b) and the effect of the hrCCN5 was significantly impaired by concomitant treatment with CCN5-antibody (Figure 1a, lane 3 and Figure 1b).

We next sought to determine whether the addition of hrCCN5 in the mouse mammary gland culture media could also be sufficient to enhance the levels of p-ER-α in ductal epithelial cells. The studies found that treatment of hrCCN5 (250 ng/ml) in the culture media resulted in profound increase of p-ER-α protein levels in the ducts/lobules and ductal epithelial cells within 7 days of treatment (Figures 1c and d). Collectively, these results indicate that CCN5 is required to regulate the expression of functionally active ER-α in both human and mouse normal mammary epithelial cells and this expression can be mediated via an autocrine-paracrine-signaling signaling loop.

### Conditional overexpression of CCN5 results in activation of ER-α in mammary epithelial cells in genetically engineered mouse model (GEMM)

**Figure 2:**
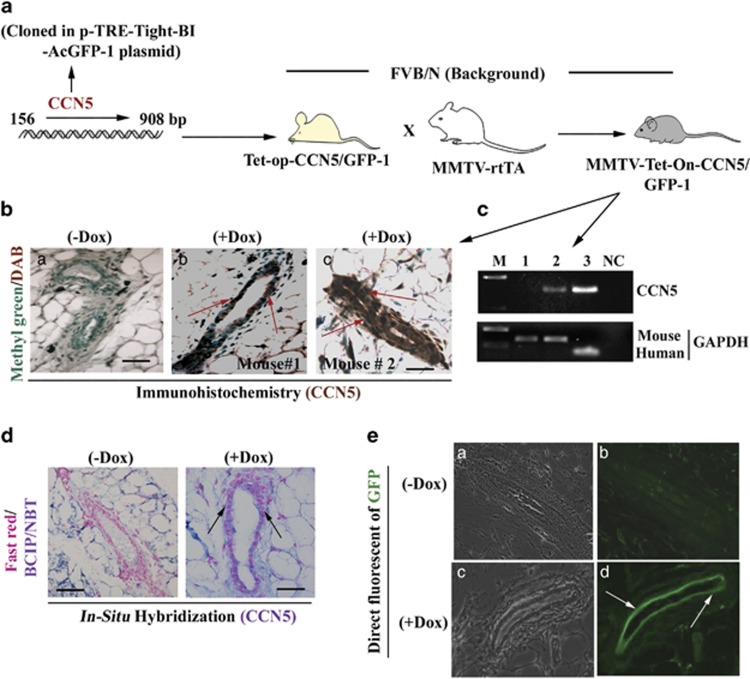
Generation of conditional transgenic mice bearing CCN5 and GFP transgenes overexpressed in mammary epithelial cells by doxycycline (Dox) treatment. (**a**) A diagram depicts the generation of CCN5-transgenic mouse (MMTV-Tet-On-CCN5/GFP-1), in which endogenous allele of human CCN5 is conditionally activated in the mammary glands by doxycycline (Dox) treatment. (**b**) Immuno-histochemical localization of CCN5 (DAB) in the ducts of mammary grands from Dox-untreated (a) and Dox-treated (b–c) transgenic mice. Methyl green was used as counter staining. Scale bars, 100 μm. Red arrows indicate the CCN5 expression. (**c**) PCR with reverse transcription analysis of CCN5 in the RNA harvested from mammary glands from Dox-untreated and treated mice. M: molecular markers. 1: untreated gland, 2: Dox-treated and 3: MCF-7 and NC: negative control. GAPDH is used as loading controls. (**d**) Localization of CCN5 mRNA expression (BCIP/NBT) in the mammary ducts from Dox untreated (−Dox) and Dox-treated (+Dox) CCN5-transgenic mice using *in situ* hybridization. Scale bars, 100 μm. The arrows indicate the CCN5 expression. (**e**) Detection of direct fluorescence of GFP in the ducts of mammary glands from Dox untreated (−Dox) and Dox-treated (+Dox) CCN5-transgenic mice. (a and c) The examples of bright fields in Dox untreated and treated samples, and (b and d) the examples of GFP-fluorescence in Dox untreated and treated samples.

**Figure 3:**
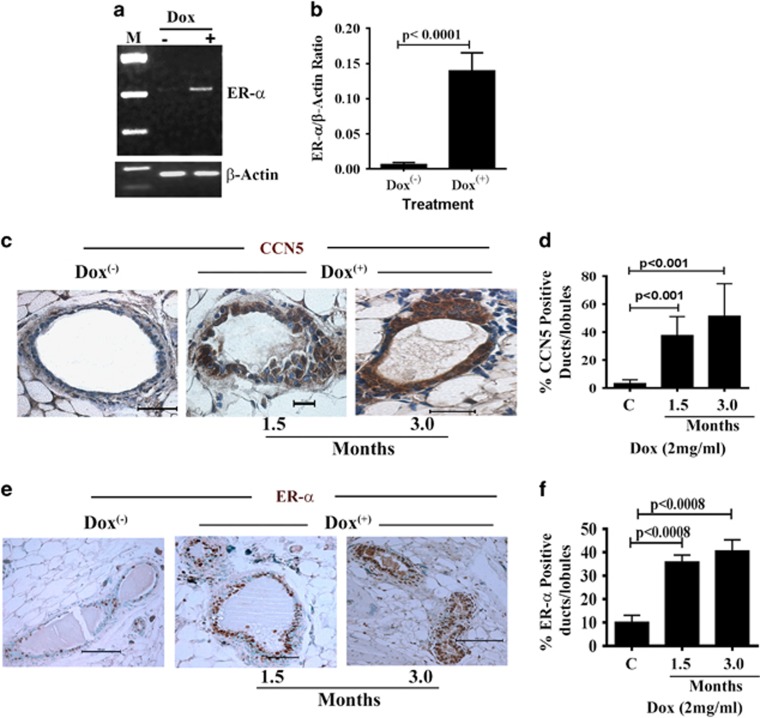
Conditional activation of CCN5 promotes ER-α expression in mammary epithelial cells of CCN5 transgenic mice. (**a** and **b**) Representative PCR with reverse transcription (**a**) and quantification (**b**) of ER-α in the RNA harvested from mammary glands from Dox-untreated and treated mice. M: molecular markers. (−): untreated gland, (+): Dox-treated. All data represent means ±s.e.m. of three independent experiments. *P*-values were calculated using two-tailed unpaired Student’s *t*-test. (**c** and **d**) Immunohistochemical localization (**c**) of CCN5 protein in the mammary glands of untreated and Dox-treated mice. Scale bars, 100 μm. The bar graph (**d**) represents the quantitative estimation of CCN5-positive ducts/lobules in Dox- treated and untreated mouse mammary glands. Data are presented as mean±s.e.m. (*n*=5 mice). (**e** and **f**) Immunohistochemical localization (**e**) of ER-α in the mammary glands of untreated and Dox-treated CCN5-transgenic mice. Scale bars, 100 μm. The bar graph (**f**) represents the quantitative estimation of ER-α-positive cells in the ducts and lobules of Dox-treated and -untreated mouse mammary glands. Data are presented as mean±s.e.m. (*n*=5 mice).

To determine the *in vivo* relevance of the above findings, we developed a doxycycline-inducible (Dox-inducible)-CCN5 transgenic mouse model (MMTV-rtTA-Tet-On-CCN5/GFP), in which CCN5 is expressed in the mouse mammary epithelial cells upon a derivative of tetracycline, doxycycline (Dox) treatment but not in other organs (Figure 2 and Supplementary Table S1). We choose five transgenic lines that exhibited approximately 4–5 × expression of CCN5 (both RNA and protein level) in the mammary epithelial cells of the cohorts with same estrous cycle upon Dox (2–4 mg/ml)-treatment for 30 days (Figures 2b and b–e and Supplementary Table S1). We then audited ER-α expression in the mammary ductal epithelial cells of the cohorts (*n*=10) following Dox treatment for different time points (that is, 1–3 months). The PCR with reverse transcription analysis found that Dox-mediated upregulation of CCN5 resulted in a dramatic increase in mRNA level of ER-α in mammary glands (Figures 3a and b, upper panel). Consistent with PCR with reverse transcription data, immunohistochemical analyses found a significant number of CCN5- and ER-α-immuno-positive ducts and lobules in the Dox-treated mammary glands of transgenic mice (10 out of 10) compared to untreated glands of transgenic mice (Figures 3c–f). The mammary ducts/lobules of Dox-treated mice exhibits overexpression of GFP, a surrogate marker of conditional expression of a gene by Dox-treatment (Figure 2e, lane d) Taken together, these data indicate that gain of CCN5 signaling activates ER-α in human and mouse normal mammary epithelial cells.

### CCN5 promotes ER-α expression in human breast cancer cells

**Figure 4:**
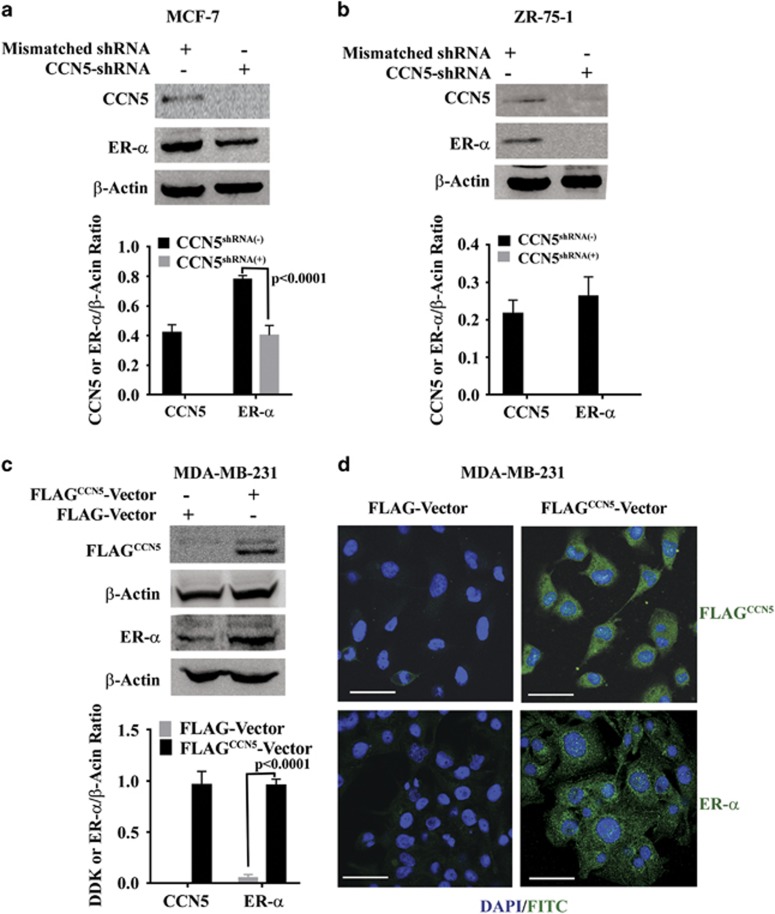
CCN5 increases ER-α protein levels in breast cancer Cells. (**a** and **b**) Representative western blots of CCN5 and ER-α in cell lysates of ER-α-positive BC cell lines MCF7 (**a**) and ZR-75-1 (**b**) transfected with scrambled shRNAs or CCN5-specific shRNAs. The bar graph represents the relative protein expression levels of CCN5 and ER-α with respect to β-actin (loading control). Data are presented as mean±s.e.m. of at least three independent experiments. *P*-values were calculated using two-tailed unpaired Student’s *t*-test. (**c**) Representative western blots of ER-α in cell lysates of CCN5-FLAG (FLAG^CCN5^) tag transfected or vector (FLAG) -transfected MDA-MB-231 cells. CCN5 levels were detected with anti-FLAG antibody. The bar graph represents the relative protein expression levels of CCN5 and ER-α with respect to β-actin (loading control). Data are presented as mean±s.e.m. of three independent experiments. *P*-values were calculated using two-tailed unpaired Student’s *t*-test. (**d**) A representative photographs of immunofluorescence using anti-FLAG tag (upper panel, green) and anti-ER-α (lower panel, green) antibodies to show increase of ER-α expression in CCN5-Flag transfected MDA-MB-231 cells compared to empty vector-transfected cells. DAPI is used to stain the nuclei (blue) and FITC-labeled secondary antibodies (green) are used for staining the antigens. Scale bars, 200 μm.

To test whether CCN5 regulates ER-α expression and activity in BC cells, we investigated the effect of CCN5 ablation by shRNA on ER-α expression in ER-α-positive MCF-7 and ZR-75-1 BC cells. In parallel, we also investigated the impact of ectopic expression of CCN5 on ER-α expression and activity in ER-α-negative MDA-MB-231 BC cells. Both studies showed that CCN5 is a positive regulator of ER-α in BC cells, as knocking down CCN5 drastically reduces the ER-α expression in MCF-7 cells and ZR-75-1 cells (Figures 4a and b), while transfection of expression vectors containing DDK-tagged-CCN5 induces ER-α expression in MDA-MB-231 cells (Figures 4c and d). To test the specificity of the role of CCN5 protein in this model, we determined if CCN6/WISP3 plays any role in regulation of ER-α in TNBC cells. To do so, MDA-MB-231 cells were treated with hrCCN5 protein (250 ng/ml) or hrCCN6 protein (250 ng/ml) for 48 h and ER-α mRNA levels were determined. As expected, ER-α mRNA expression was significantly increased in hrCCN5-treated samples, while this effect remained undetected in hrCCN6-treated samples (Supplementary Figure S1).

### CCN5 regulates ER-α expression at the transcription level in BC cells via interacting with integrins

**Figure 5:**
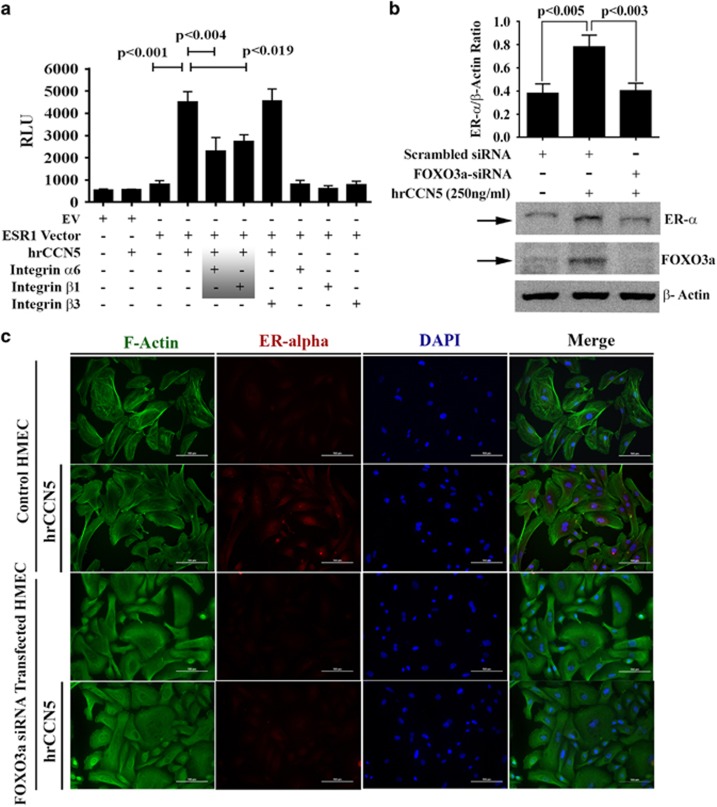
Integrins α6β1 and FOXO3a are required for CCN5-mediated regulation of ER-α expression in BC cells. (**a**) ER-α luciferase assays. MDA-MB-231 cells were transfected with empty construct or ER-α-Luc promoter construct for 48 h followed by hrCCN5 protein (250 ng/ml) treatment or vehicle alone for a further 48 h and also in the presence or absence of different neutralizing antibodies of integrins. A luciferase assay was performed using luciferase assay kits. EV, empty vector, and ESR1, ER-α promoter vector. Data are presented as mean±s.e.m. of eight independent experiments. *P*-values were calculated using two-tailed unpaired Student’s *t*-test. (**b**) Representative western blots and quantification of ER-α in cell lysates of FOXO3a-siRNA-transfected or scrambled siRNA-transfected MDA-MB-231 cells. Data are presented as mean±s.e.m. from triplicate experiments. *P*-values were calculated using two-tailed unpaired Student’s *t*-test. (**c**) Immunofluorescence analysis for the detection of the effect of FOXO3a ablation on ER-α expression in HMEC cultured in the presence or absence of hrCCN5 (250 ng/ml). Scale bar, 100 μm. See also Supplementary Figure S1.

To reveal the mechanism whereby CCN5 induces ER-α expression, we investigated the effect of CCN5 on ER-α promoter. To do so, a reporter plasmid (pLightSwitch_Prom) containing a sequence of the human ER-α (ESR1) promoter^34^ was transfected into MDA-MB-231 cells for 48 h and then treated with hrCCN5 (250 ng/ml) or vehicle alone for a further 48 h. CCN5 stimulation substantially increased luciferase activity in ESR1-prom-transfected cells compared to untreated cells (vector-transfected cells or ESR1-prom transfected cells) (Figure 5a, lane 2 or 3 vs 4), suggesting CCN5 regulates ER-α at the transcription level. Ironically, the hrCCN5-untreated ESR1-transfected cells (lane 3) also exhibit significant induction of luciferase activity compared to empty reporter vector transfected cells (lanes 1 and 2) indicating that some serum component(s) could be responsible for this induction.

Because members of the CCN family including CCN5 pursue many differential activities through their direct binding to integrins including integrins α6, β1 and β3,^28, 35^ we then tested whether the CCN5-induced ER-α promoter activity can be mediated through aforementioned integrins. To do so, promoter-transfected MDA-MB-231 cells were treated with different antibodies of integrins for 24 h followed by hrCCN5 protein, as indicated in Figure 5a. We found that the antibodies against integrins α6 and β1 significantly impaired the hrCCN5 effect on ER-α-promoter activity, while antibody against integrinβ3 was unable to interfere in CCN5-mediated induction of ER-α. The treatment of integrin antibodies alone has no detectable effect on ER-α-promoter-mediated luciferase activities.

### Akt-Foxo3a-signaling is an addition critical component for the regulation of ER-α by CCN5

Previously, it has been reported that transcription factor FOXO3a (formerly known as FKHRL-1) is a regulator of ER-α gene transcription and the FOXO3a mediated regulation of ER-α can be repressed by PI3K/Akt-signaling pathway.^36^ Recently, our studies have shown that CCN5 enhances FOXO3a expression via suppressing Akt signaling in TNBC cells.^28^ Thus, we studied to determine if CCN5-induced upregulation of ER-α is mediated through FOXO3a in BC cells. MDA-MB231 cells were transfected with a FOXO3a-siRNA that suppresses FOXO3a about 70% or scrambled siRNA (Figure 5b). Transfected cells were grown in the presence or absence of hrCCN5 for 48 h and cell extracts were prepared and subjected to immunoblotting for ER-α. As expected, CCN5 treatment resulted in increase in the ER-α level (Figure 5b, lane 2), while decreasing FOXO3a expression by siRNA resulted in significant reduction in the ER-α level and it reaches to the basal level (Figure 5b, lane 3). Similar effect was observed in normal mammary epithelial cells. We find by using immunofluorescence labeling studies that ablation of FOXO3a by RNAi in HMEC (Supplementary Figure S3), markedly reduces the effect of CCN5 on ER-α expression (Figure 5c). Taken together, these studies indicate that CCN5-induced upregulation of ER-α is mediated via FOXO3a.

Since FOXO3a is negatively regulated by Akt signaling,^36^ the ability of CCN5 to regulate Akt phosphorylation was determined using western blotting as well as in-cell-western blotting. Consistent with our previous findings,^28^ Akt phosphorylation levels were significantly reduced in MDA-MB-231 cells upon CCN5 treatment (Supplementary Figure S2). Thereby, we can anticipate that suppression of Akt activity by CCN5 is critical to increase ER-α-activity via FOXO3a in BC cells

#### CCN5-induced expression of ER-α is functionally active

**Figure 6:**
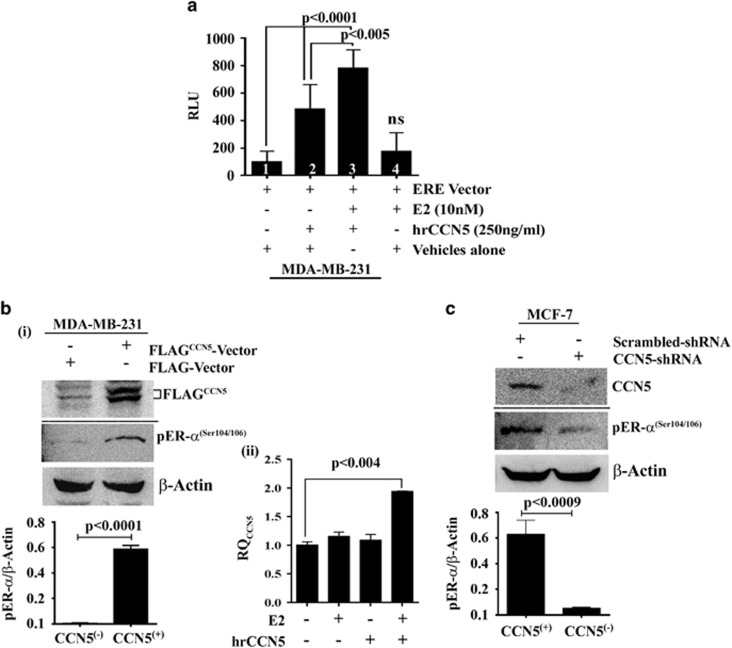
CCN5-induced expression of ER-α is functionally active in BC cells. (**a**) Functional luciferase assay. MDA-MB-231 cells were transfected ERE vectors or vector alone for 48 h followed by E2 or hrCCN5 or combination treatment for a further 48 h and luciferase activity was measured. Data are presented as mean±s.e.m. of eight independent experiments. *P*-values were calculated using two-tailed unpaired Student’s *t*-test. (**b**i) ER-***α*** phosphorylation change in MDA-MB-231 cells following CCN5 transfection. Representative western blots of FLAG^CCN5^ and phosphor-ER-α (Ser104/106) in cell lysates of FLAG-vector (FLAG) or FLAG-CCN5-vector (FLAG^CCN5^) transfected MDA-MB-231 cells. The Bar graph represents the relative protein expression levels of p-ER-α with respect to β-actin (loading control). Data are presented as mean±s.e.m. of at least three independent experiments. *P*-values were calculated using two-tailed unpaired Student’s *t*-test. (ii) Induction of CCN5 mRNA expression by E2-treatment in MDA-MB-231 cells exposed to hrCCN5. The bar graph represents the relative mRNA expression levels (RQ) of CCN5 in MDA-MB-231 cells exposed to E2 in the presence or absence of hrCCN5 for 48 h. Data are presented as mean±s.e.m. of at eight independent experiments. *P*-values were calculated using two-tailed unpaired Student’s *t*-test. (**c**) ER-*α* phosphorylation change in MCF-7 cells following CCN5 ablation by shRNA. Representative western blots of CCN5 and phosphor-ER-α (Ser104/106) in cell lysates of scrambled shRNA or CCN5-shRNA transfected MCF7 cells. Bar graph represents the relative protein expression levels of p-ER-α with respect to β-actin (loading control). Data are presented as mean±s.e.m. of at least three independent experiments. *P*-values were calculated using two-tailed unpaired Student’s *t*-test.

To test whether induced expression of ER-α by CCN5 in TNBC cells is functionally active, we performed *in vitro* ER-α-estrogen-response element (ERE) binding reporter assay in the presence or absence of ligand E2 in MDA-MB-231 cells. MDA-MB-231 cells were transfected with pLightSwitch_Prom vectors containing ERE and then cells were treated with E2 (10 nm) or hrCCN5 alone or in combination for 48 h and luciferase activities were measured. As shown in Figure 6a, E2-treatment significantly promotes luciferase activity in hrCCN5-treated cells (lane 3) as compared to vehicle-treated (lane 1) and only estradiol-treated cells (lane 4). Thereby, this study indicates that the hrCCN5-induced-ER-α is functionally active to form E2-ER complexes and bind to ERE leading to gene transcription. Since the luciferase-activity also markedly elevated in hrCCN5-treated cells in the absence of E2-treatment (lane 2), we can anticipate that other factors such as phosphorylation of ER-α by CCN5 may promote ER-α-ERE binding *in vitro* in the absence of ligand.^37, 38, 39, 40^

The primary function of phosphorylation-dephosphorylation of ER-α is effective regulation of the functional activity of ER-α by controlling ER-α-ERE binding to modulate expression of target genes.^37, 38, 41^ The goal of our studies was to determine the effect of ectopic expression of CCN5 in MDA-MB-231 cells or the shRNA-based depletion of CCN5 in MCF-7 cells on phospho-ER-α (Ser104/106), a surrogate marker of functionally activated ER-α.^42^ The studies found that the level of phospho-ER-α (Ser104/106) was significantly increased in CCN5-transfected MDA-MB-231 cells while markedly reduced in CCN5-depleted MCF-7 cells compared to their corresponding controls (Figures 6bi and c). Together, these studies indicate that the CCN5-induced upregulation of ER-α in TNBC cells are functionally active. Further corroborate the above results; we determined whether E2-treatment is able to activate CCN5 transcription in MDA-MB-231 cells in which ER-α was activated by hrCCN5 treatment. To do so, MDA-MB-231 cells were treated with E2 (10 nm) in the presence or absence of hrCCN5 (250 ng/ml) for 48 h and CCN5 mRNA levels were determined using quantitative PCR. CCN5 mRNA expression was significantly elevated in E2+hrCCN5-treated cells as compared to untreated, E2 treated and hrCCN5 alone treated samples (Figure 6bii).

### Depletion of CCN5 signal desensitizes ER-α-positive BC cells to estrogen and antiestrogen

**Figure 7:**
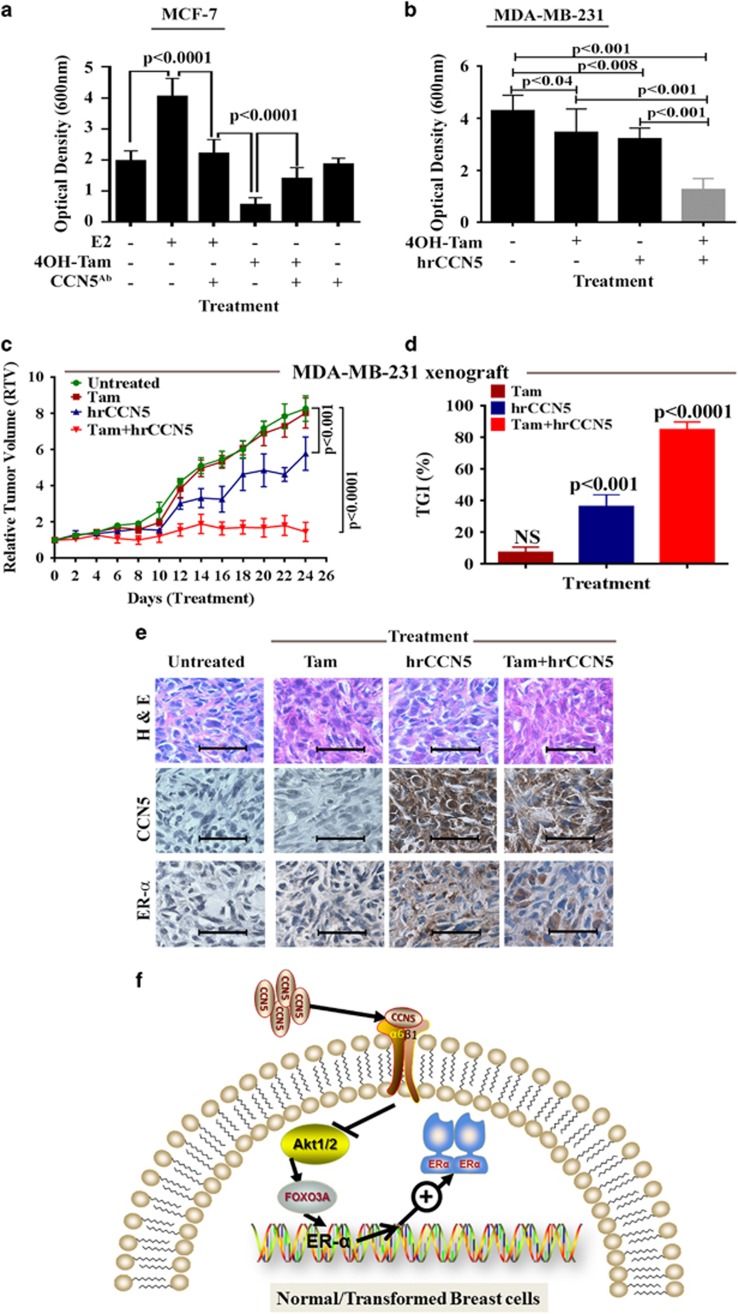
CCN5 and Tamoxifen exhibits additive effect on TNBC cell growth *in vitro*and*in vivo.*(**a**,**b**) Cell growth assay. MCF7 (**a**) and MDA-MB-231(**b**) cells treated with 17β-estradiol (E2, 10 nm) or 4-hydroxy-Tamoxifen (4OH-Tam, 1 μm) or both in the presence or absence of CCN5 antibody (CCN5^Ab^, 500 ng/ml) or CCN5 recombinant protein (hrCCN5, 250 ng/ml) for 48 h and cell viability was determined using crystal violet assay. Data are presented as mean±s.e.m. of eight independent experiments. *P*-values were calculated using one way analysis of variance and two-tailed unpaired Student’s *t*-test. (**c**,**d**): Additive antitumor efficacy of CCN5 and Tam in xenograft model. MDA-MB-231 xenograft female mouse model (*n*=5) was treated with Tam (oral), hrCCN5 [intratumoral (it) injection] or combination three times a week for 24 days. Growth curve was measured using RTV three times per week and %TGI was measured as end point tumor growth using analysis of variance and two-tailed unpaired Student’s *t*-test. Data are presented as mean±s.e.m. of five animals. (**e**) Hematoxylin and eosin (h and e) and immunohistochemical localization of ER-α and CCN5 in tumor samples of MDA-MB-231 xenograft model. Scale bar, 100 μm. (**e**) Model of CCN5-induced ER-**α** expression in BC cells. Upon suppression of Akt signaling by CCN5-α6β1intigrins axis, FOXO3a activates. The activated FOXO3a promotes ER-α expression, thereby facilitating hormonal action in ER-α-negative BC cells.

For further confirmation of the involvement of CCN5 in activation and functional response of ER-α in BC cells, we determined whether blocking CCN5 activity by CCN5-specific antibody treatment reduces the proliferative effect of E2 or impede the effect of 4OH-Tam (4-hydroxy-tamoxifen) on MCF-7 cells. Consistent with previous works,^13, 43, 44, 45, 46^ E2 (10 nm) treatment for 48 h, significantly increased MCF-7 cell viability, and this effect of E2 was significantly blocked when cells were pre-treated for 48 h with CCN5-antibody (CCN5^Ab^) followed by concomitant treatment with E2 and CCN5^Ab^ (Figure 7a). Moreover, CCN5^Ab^ treatment also reduced the cytotoxic effect of 4OH-Tam (10 μm) in these cells but was unable to reduce downright. The partial rescue of MCF-7 cells from catastrophic effect of 4OH-Tam can also be seen in shRNA-mediated CCN5-knockout MCF-7 cells (Supplementary Figure S4). Tam exerts both receptor (ER-α)-dependent and independent pathways to kill BC cells.^47^ Thus, CCN5 ablation, which blocks ER-α expression, may partially promote desensitization to Tam. These results further support the proposal that CCN5 is an essential molecule in BC cells to maintain the ERα expression and activity.

### CCN5 treatment sensitizes TNBC cells to tamoxifen

The aforesaid collective studies demonstrate strongly that CCN5-signaling is critical for ER-α-regulation in normal and neoplastic breast cells and suggest its implication in therapeutic benefit of TNBC. Thereby, we investigated the impact of 4OH-Tam (10 μm) on MDA-MB-231 TNBC cell growth in the presence or absence of hrCCN5 (250 ng/ml). Consistent with previous studies, the cell viability studies revealed a minimum growth inhibitory effect of Tam (10 μm for 48 h) on MDA-MB-231 cells (Figure 7b). An additive effect was observed when MDA-MB-231 cells were treated with 4OH-Tam in the presence of hrCCN5 for 48 h (Figure 7b), and this effect can be restrained by ER-α-RNAi treatment (data not shown). A growth inhibition of MDA-MB-231 cells was also documented in treatments containing only hrCCN5 (Figure 7b). Collectively, the studies suggest that CCN5 boosts Tam action in these cells through the activation of ER-α.

The progressive amplification of the response and possible therapeutic potential of Tam-hrCCN5 treatment was further investigated in a subcutaneous human TNBC xenograft MDA-MB-231 mouse model (Figures 7c and d). In this xenograft model, while treatment of Tam alone led no significant response, combination of hrCCN5 and Tam treatment exhibits significant inhibition of relative tumor volume (RTV) and increase in percent end point tumor growth inhibition (%TGI). The TGI enhancement was associated with ER-α over expression in the tissue samples (Figure 7e). CCN5 protein, which is normally absent in MDA-MB-231-tumor xenograft, was detected in tumor samples of treated groups implicating the bioavailability of hrCCN5 protein in the target sites (Figure 7e). Moreover, no morbidities or body weight loss were detected in these group of animals indicating undetectable or controllable toxicity of these treatment groups (Supplementary Figure S5).

## Discussion

These studies provide two significant discoveries. First, these studies prove that CCN5 promotes ER-α expression and endorses its activity in normal breast ductal and lobular epithelial cells and BC cells. CCN5-mediated induction of ER-α expression, at least in BC cells, is a transcriptional regulation through the integrin α6β1-FOXO3a pathway and possibly via suppressing Akt-signaling. The involvement of same mechanism in regulation of CCN5-mediated ER-α-expression in normal mammary epithelial cells is also anticipated as FOXO3a ablation blocks CCN5-induced ER-α expression in HMEC cells. Second, we report that restored ER-α by CCN5 is functionally active and makes TNBC cells sensitive to Tamoxifen (brand name Nolvadex and others).

More than 60% of human BC patients’ samples are immunoreacted with the antibodies of ER-α, which is an important bio-marker and portends good prognosis as they are less aggressive and respond to hormonal therapy such as Tam.^48, 49, 50^ However, in reality, this is not always the case because only two-thirds of the advanced ER-positive BC patients respond to Tam.^48, 51^ Non-responding tumors either express no ER-α during diagnosis, were initially positive but eventually lose ER-α or are dysfunctional during invasive growth and perhaps become hormone independent and endocrine therapy resistant.^13, 48, 51, 52^ Collectively, it is manifested that the disappearance of ER-α in BC cells is one of the vital causes of the aggressive behavior of this disease and prone to relapse.^53^ However, the regulation of ER-α expression and activity in normal and cancer cells in breast are still poorly defined.

Multiple theories have been proposed regarding the regulation of ER-α in various ER-positive cells. The epigenetic concept, which is still debatable, indicates that ER-α silencing, stability or turnover is a complicated event and is caused by either transcriptional, post-transcriptional or both modifications. These include aberrant methylation of CpG islands in the 5′-regulatory sequence of ER-α gene,^48, 52, 54^ phosphorylation,^55, 56, 57^ acetylation,^58^ SUMOylation^59^ and ubiquitination.^60, 61^ The post-transcriptional modifications of ER-α, particularly phosphorylation, acetylation and SUMOylation may crosstalk with ubiquitination process^62^ through Forkhead box K2 (FOXK2) transcription factor and ubiquitin E3 ligase BRCA1/BARD1 complex.^45^ Additional concept of restraining ER-α implicate the role of a transcription factor FOXO3a in direct regulation of ER-α gene transcription and is suppressed by the Her-2/neu/phosphatidylinositol 3-kinase/Akt signaling pathway.^36^ Despite having different mechanistic approaches, it is still unclear which autocrine-paracrine signaling regulates ER-α and how it implies the process. Our findings within normal and tumor milieu in tissue culture and mouse model indicates a strong link between CCN5 signaling and ER-α expression. We found that induced expression of CCN5 or treatment of CCN5 protein markedly elevated ER-α expression in normal human and murine breast epithelial cells or human BC cells devoid of ER-α. In contrast, suppression of CCN5 by shRNA or antibody treatment reduces ER-α expression in human ER-α-positive BC cells. The studies have also demonstrated that the upregulated ER-α is functionally active in both normal and transformed cells. Mechanistically, based on current and our previous work on BC cells,^28^ CCN5 interacts with integrin α6β1 to suppress the PI3K/Akt-signaling which then activates FOXO3a. Activated FOXO3a in turn upregulates activated form of ER-α (Figure 7f), and enhances the response of estrogen or estrogen antagonist. Because CCN5 is an estrogen response gene in ER-α-positive BC cells^25^ and present studies showed that the estradiol-treatment is able to activate CCN5 transcription in hrCCN5-treated TNBC cells (Figure 6), we conceived the possibility of a autocrine-paracrine feedback signaling loop between CCN5 and ER-α for the regulation of these molecules in both normal and cancer epithelial cells of mouse and human breasts with different functional perspectives.

ER-α is required for the Tam action as an antagonist of estrogen to prevent BC cell growth. However, as indicated earlier, due to lack of ER-α or functional inactivation of ER-α via complex epigenetic paradox^45, 56, 57, 58, 60, 63, 64^ or other mechanisms, some BC cells become Tam-resistant,^13, 48, 51, 52, 64^ resulting in an enormous therapeutic burden. The present *in vitro* and *in vivo* studies, in addition to mechanistic perceptions, suggest a potential therapeutic significance by providing a mechanism-based rationale for combination therapy of Tam and CCN5 in TNBC. Since the CCN5 activator is not currently available, our goal is to find a new molecule as an activator of CCN5. In addition, this study suggests that hrCCN5 may be readily tested in preclinical trials.

In summary, the findings presented a new layer of complexity to the mechanisms by which ER-α is activated in normal and transformed breast epithelial cells. CCN5 may become a prognostic marker and therapeutic target for TNBC and designing CCN5 activators is a promising approach to control TNBC growth.

## Materials and methods

### Ethics statement

Animal protocols were approved by the KCVAMC Animal Care and Use Committee, in accordance with the AAALAC animal care guidelines, and NIH Guide for the Care and Used of Laboratory Animals. Mice have been monitored daily and euthanized when displaying excessive discomforts. The mice were fed regular commercial mouse diet (without tetracycline) with a 12 h light-dark cycle.

### Experimental animals

Wild-type FVB/N mice (male and female) were purchased from Taconic Biosciences (Hudson, NY, USA) and housed in the Kansas City Veterans Administration Medical Center (KCVAMC) animal care facilities. MMTV-rtTA transgenic male and female mice were obtained as a generous gift from Dr Chodosh’s laboratory. CCN5/GFP conditional transgenic mice were generated at the University of Kansas Medical Center animal facilities. CCN5 conditional transgenic mice were obtained from the mating between MMTV-rtTA transgenic and CCN5/GFP mice at the animal care facilities of KCVAMC.

### Chemicals and antibodies

All the chemicals and drugs including 4-hydroxy-tamoxifen (Tam or 4OH-Tam; Cat#H7904) and 17β-estradiol (E2; Cat#E2257) were purchased from Sigma-Aldrich (St Louis, MO, USA). CCN5 human recombinant protein (hrCCN5; Cat#120–16) was purchased from PeproTech (Rocky Hill, NJ, USA). Doxycycline (Dox) was purchased from Takara Bio (Mountain View, CA, USA, Cat # 631311). Antibodies for western blot analysis, immunofluorescence, immunohistochemical staining were purchased from following vendors: Anti-ERα (Cell Signaling, Danvers, MA, USA; Cat#13258 and Abcam, Cambridge, MA, USA; Cat#ab32063), anti-p-ER-α (Cell Signaling, Cat#9924), Anti-CCN5 (Abcam, Cat#ab38317), anti-Akt (Cell Signaling, Cat#4691), anti-p-Akt (Cell Signaling, Cat # 9271), mouse Anti-FLAG/DDK (Origene, Rockville, MD, USA, Cat#TAG0011), mouse anti-β-actin (Sigma, Cat#A3853), anti-Integrins α6 (Millipore, Billerica, MA, USA, Cat#MAB1378) and anti-Integrins β1 (Millipore, Cat#MAB1987Z). The authentication certificates for all these chemicals, drugs and antibodies were provided by these companies. The fresh working solutions of the chemicals and drugs were prepared once a month to guarantee effectivity.

### Construction of the targeting vector

The objective of this study was to generate a mouse with the human CCN5 gene under the control of Tet-operator (tetracycline controlled promoter). To do so, the coding sequence of human CCN5 (765 bp) was cloned to the multiple cloning sites positioned downstream of the Tet promoter in a pTRE-Tight BI-AcGFP-1 vector (Takara Bio, Cat#631066). This vector contains a modified TRE and minimal CMV promoter that provide better control of gene expression by eliminating leaky transgene expression in the absence of inducer. Moreover, this vector also contains a green fluorescence gene sequence (Ac-GFP1) that can be regulated under the control of tetracycline). Initially, PRK5-hCCN5 vector was obtained from Dr Pennica (Genentech Inc., San Francisco, CA, USA). CCN5 cDNA fragments were amplified by PCR using KpnI and NheI restriction sequences tagged forward and reverse primers, respectively. The PCR generated fragments were cloned into the KpnI and NheI restriction sites of the multiple cloning sites of the vectors. The recombinant clones were verified by restriction digestion analysis, PCR with human CCN5 primers and sequencing. After the preparation of the construct, the founder line was generated by injecting the linearized construct into fertilized oocytes that was harvested from super-ovulated FVB/N mice. All founders will be genetically identical because of their (FVB/N) fully inbred genetic background. All the resulting pups (Tet-op-CCN5) were screened for the presence of transgene using primers within pTet-Splice.

### Generation of CCN5 conditional transgenic mice

CCN5-transgenic mice were bred with MMTV-rtTA mice to generate MMTV-rtTA/Tet-op-CCN5-conditional transgenic mice. PCR screening was used by specific primers from tail DNA to evaluate the mice for all three transgenic elements. After the confirmation, we determined whether CCN5 expressed conditionally in a mammary epithelial specific in a dox-dependent manner (time and dose). To test this, Dox (2–4 mg/ml in 10% sucrose) was added twice a week in the drinking water as described earlier^65, 66^ for different time points (45 and 90 days). Breast tissues were harvested and different molecular and histological parameters were evaluated.

### Cell lines, culture conditions and transfection

MCF-7, ZR-75-1 and MDA-MB-231 cell lines were obtained from American Type Culture Collection (ATCC, Manassas, VA, USA) and maintained in Dulbecco’s modified essential medium supplemented with 10% FBS and antibiotics. HMECs were obtained from LONZA (MD, USA) and maintained in MEBM (mammary epithelial basal medium) with growth factors. Cell lines were grown in a humidified incubator with 5% CO_2_ at 37 °C. The cell lines used in these studies were authenticated as a standard quality control measure at the beginning and end of the project and when banking frozen materials for later use. The authentications of these cells were performed using STR profiling (Short Tandem Repeat analysis of DNA) and were checked every two months for mycoplasma contamination.

Transfection was performed with the Neon Transfection System (Thermo Fisher Scientific, Waltham, MA, USA) as per manufacturer’s instruction. Briefly, ~70% confluent cells were trypsinized, washed with 1 × PBS and then suspended in Buffer R and mixed with plasmid DNA (5–10 μg). The cell-DNA mixtures were electroporated for transfection at selected voltage pulse using Neon Transfection System.

#### Cell viability assay

Cell viability assay was performed according to our previous method.^67^ Cells were seeded in quadruplicates in 96-well plates. Approximately 60–70% confluent serum-deprived MCF-7 cells were treated with CCN5 antibody (500 ng/ml) for 48 h and followed by E2 (10 nm), Tam (10 μm) concomitant treatment for another 48 h. MDA-MB-231 cells were treated with Tam (10 μm) and hrCCN5 (250 ng/ml) together for 48 h. Cellular viability was measured using crystal violet-based cell viability assay. The viability studies were also carried out in CCN5-shRNA transiently transfected MCF-7 cells. More-detailed procedures can be found in the Supplementary Information.

### RT-PCR analysis

Cytoplasmic RNAs were extracted mouse breast tissue samples using the Trizol extraction procedure as previously described^68^ and were subjected to reverse transcription using a PCR with reverse transcription RNA amplification kit (Perkin Elmer, Waltham, MA, USA). PCR was amplified with human CCN5 and mouse and human GAPDH (for equal loading) -specific primers. The sequences of the primers were: human CCN5, 5′-CCTACACACACAGCCTATATC- 3′ (Forward), and 5′-CCTTCTCTTCATCCTACCC- 3′ (Reverse), ER-α, 5′TTCTCCCTTTGCTACGTCAC-3′ (Forward) and 5′ATCGCTTTGTCAACGACTTC3′ (Reverse) and mouse GAPDH, 5′CTGCTGTCTTGGGTG CAT TGG-3′ (Forward) and 5-CTCGGCTTGTCACATCT–3′ (Reverse).

### Immunohistochemistry

The immunohistochemical staining for CCN5 was performed on formalin-fixed, paraffin-embedded tissue sections according to our previous method.^31^ The sections were imaged with a Leica photomicroscope.

### Immunofluorescence

Cells were fixed with methanol for 20 min and then permeabilized with 0.1% Triton X-100/PBS for 5 min. Samples were blocked with a ready-to-use blocking solution (Histostain kit, Invitrogen) and incubated with mouse-anti-FLAG/DDK and rabbit anti-ER-α antibody overnight at 4 °C. Cells were then stained with anti-rabbit IgG fluorescent conjugate (Alexa Flour 488, Molecular Probes, Eugene, OR, USA), and nuclei were counterstained with 4′,6-diamidino-2-phenylindole (DAPI). Cells were mounted with antifade mounting reagent (Molecular Probes), and examined by Leica confocal fluorescence microscopy.

### Detection of direct fluorescence of GFP

Direct fluorescence of GFP was detected using the previous method.^69^ Briefly, 4% formalin-fixed-paraffin-embedded mouse breast tissue sections (5 μm) from Dox-treated and untreated were deparaffinized, rehydrated in different grades of alcohol and washed with PBS. The slides were then passed through the upgrade of alcohol and mounted with mounting media and examined under a fluorescent microscope.

### Probe preparation and *in situ* hybridization

The DIG-labeled PCR-based probe preparation was same as described in previous reports.^31, 70^ Briefly, 5-μm paraffin sections were deparaffinized, hydrated and digested with proteinase K for 10 min followed by post fixation in 1% formaldehyde/1 × PBS. The slides were washed with RNAse-free distilled water for 5 min. The sections were incubated overnight at 37 °C in a humidified chamber with the DIG-labeled PCR-generated CCN5 probe (250 ng/ml). The slides were washed three times with PBS and 0.1% Tween-20. Alkaline phosphatase-conjugated anti-DIG antibodies were applied for 1 h and complexes were detected with the chromogen combination 5-bromo-4-chloro-3-indolyl phosphate and NBT. The sections were counterstained with nuclear fast red.

### Western blot analysis and antibodies

For protein analysis, cells were harvested and subjected to western blotting as previously described.^31, 71^ Signals from the blots were detected with Super Signal Ultra Chemiluminescent substrate by using Kodak ID Image Analysis software Version 3.6 (Caresteam, Rochester, NY, USA).

### Promoter assay

MDA-MB-231 cells were transfected with promoter reporter vector, pLightSwitch_LR (SwitchGear Genomics) with promoter sequence of human ESR1 (ER-α) gene or human ERE cloned in the multiple cloning site of the vector, immediate upstream to the Renilla Luciferase reporter gene. Transfected cells were grown in the presence or absence of hrCCN5 protein in OptiMEM media at a concentration of 250 ng/ml and treated with the antibodies against different integrin receptors (Cell Signaling) or left untreated. The difference of luciferase activity was measured following the protocol provided by the manufacture.

### *In vivo* treatment

MDA-MB-231 xenograft model was used for this study. Briefly, MDA-MB-231 cell (1 × 10^6^) with matrigel were injected subcutaneously into the nude mice (N=5/per experiment). Once the average tumor volume reached 80–100 mm^3^, mice were randomized 4 groups and started treatment. Tam (2.5 mg/mouse) was given orally three times a week and hrCCN5 (2 mg/Kg) injected twice a week intratumorally (it) for 27 days. For combination treatment, both Tam and hrCCN5 were given concomitantly. Tumor growth, RTV, %TGI and body weight of the mice were measured using studylog^R^ measurement tools and softwares (California, USA) three times a week and also described elsewhere.^72^ The tumor volume (TV) was estimated the formula: TV (mm^3^)=L (length) × W (width)^2^/2 The RTV was calculated using formula: RTV=*V*_d_/*V*_o_. *V*_d_ is the tumor volume of each day while V_o_ is the initial tumor volume at the beginning of the experiments. The %TGI was estimated using the formula: %TGI= [1-(RTV treated group)/RTV control group) × 100] at the final day of treatment.

### Statistical analysis

The statistical analysis was performed using the Graph Pad Prism 4 (GraphPad Software, Inc., La Jolla, CA, USA) and PASS^15^softwares, NCSS, LLC (Kaysville, UT, USA). Results are shown as mean±s.d. Means between the groups were calculated and compared among or within variants using a two-sided Student’s *t*-test. RTV and TGI rates were determined using a two-sided Student’s *t*-test, two-way ANOVA. *P-*value of <0.05 was considered statistically significant. We calculated the required sample size for *in vitro* studies using an approximate method^73^ is *n*=5–8 cultures per groups and time point, assuming comparison-wise type I error of 5% and power of 80% to detect the probability of concordance of 75%. The required number of mice, calculated is at least 10 mice per group and time point, assuming power of 85%, type I error of 5%, probability of concordance between treatment and tumor measurements of 75%, and experimental success rate of 80%. The entire studies were performed blindly by two or more investigators.

## Abbreviations

CCN5: Cysteine-rich angiogenic inducer 61 (Cyr61), connective tissue growth factor (CTGF), and Nephroblastoma Overexpressed (NOV)-5
ER-α: Estrogen Receptor-alpha
TAM: Tamoxifen
BCIP: 5-bromo-4-chloro-3′-indolyphosphate p-toluidine salt
NBT: nitro-blue tetrazolium chloride
