# Supplementary material for: CCN5/WISP-2 restores ER-∝ in normal and neoplastic breast cells and sensitizes triple negative breast cancer cells to tamoxifen
Source: Oncogenesis. 2017 May 22;6(5):e340–. doi: 10.1038/oncsis.2017.43 (PMC5569333; doi:10.1038/oncsis.2017.43)
Supplement: Supplementary Materials [file oncsis201743x1.doc]

# SUPPLEMENTAL INFORMATION

**CCN5/WISP-2 restores ER-∝ in normal and neoplastic breast cells and sensitizes triple negative breast cancer cells to tamoxifen**

Sandipto Sarkar^1, 5𝛉^ , Arnab Ghosh^1, 2𝛉^, Snigdha Banerjee^1, 2*^, Gargi Maity^1, 3^, Amlan Das^1, 2, 3#^, Melissa A. Larson^3^, Vijayalaxmi Gupta^1,2^, Inamul Haque^1,2^, Ossama Tawfik^4^, and Sushanta K. Banerjee^1, 2, 4, 5, *^

INVENTORY OF SUPPLEMENTAL INFORMATION

1. **Supplemental Data**

**Contain Supplemental Figures S1 and S3 with legends**

- **Figure S1** (related to Figure 4)
- **Figure S2** (related to Figure 5)
- **Figure S3** (related to Figure 5)
- **Figure S4** (related to Figure 7)
- **Figure S5** (related to Figure 7)
- **Table S1**


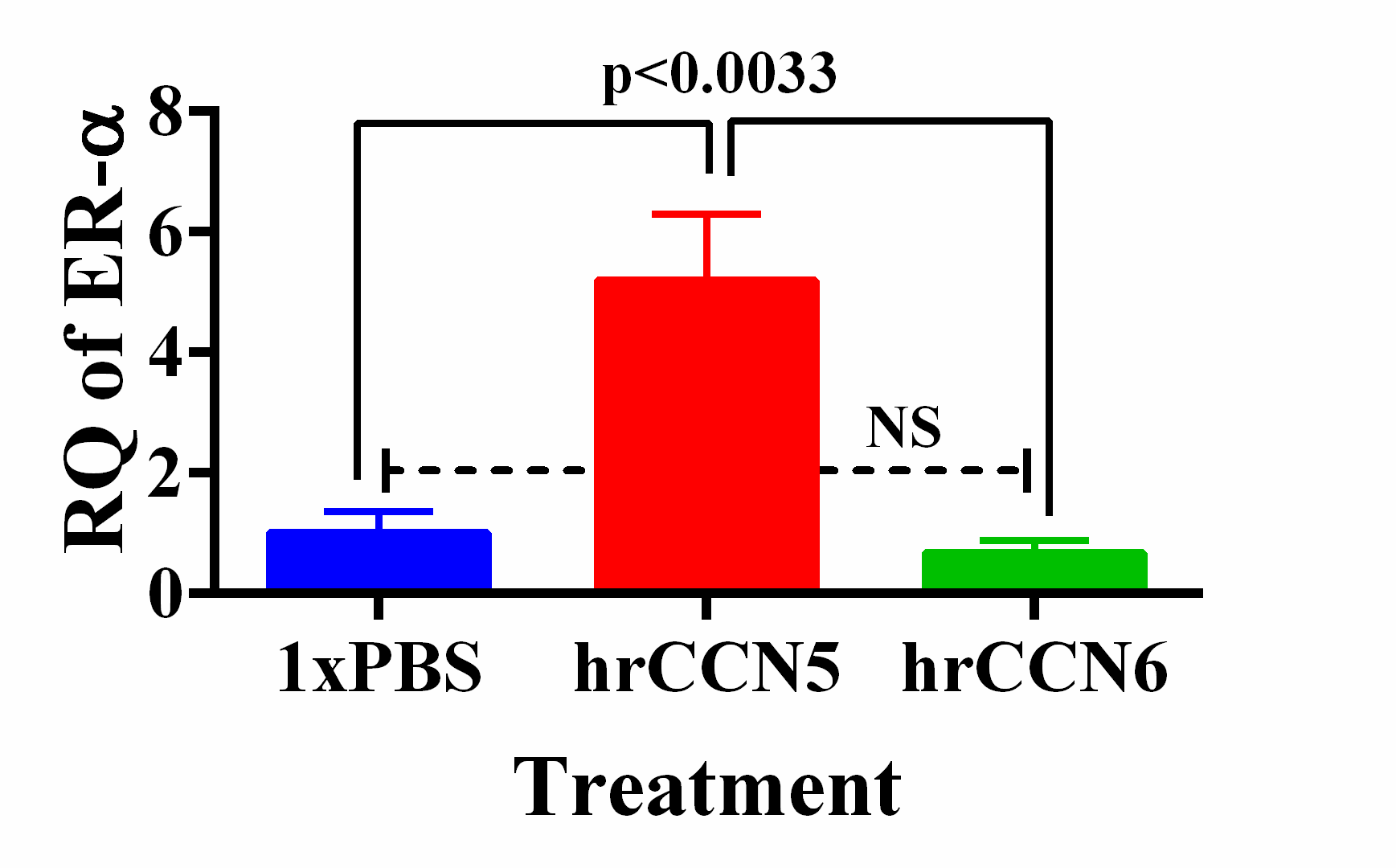


**Figure S1 (related to Figure 4): ER-𝛂-regulation by CCN5 and CCN6 protein in TNBC cells.** To test the specificity of the role of CCN5 protein in this model, we determined if CCN6/WISP3 plays any role in regulation of ER-α in TNBC cells. To do so, MDA-MB-231 cells were treated with hrCCN5 protein (250ng/ml) or hrCCN6 protein (250ng/ml) for 48h and ER-α mRNA levels were determined. All data represent means ±SEM of eight independent experiments. P values were calculated using one way ANOVA and two-tailed unpaired Student’s t-test.

**
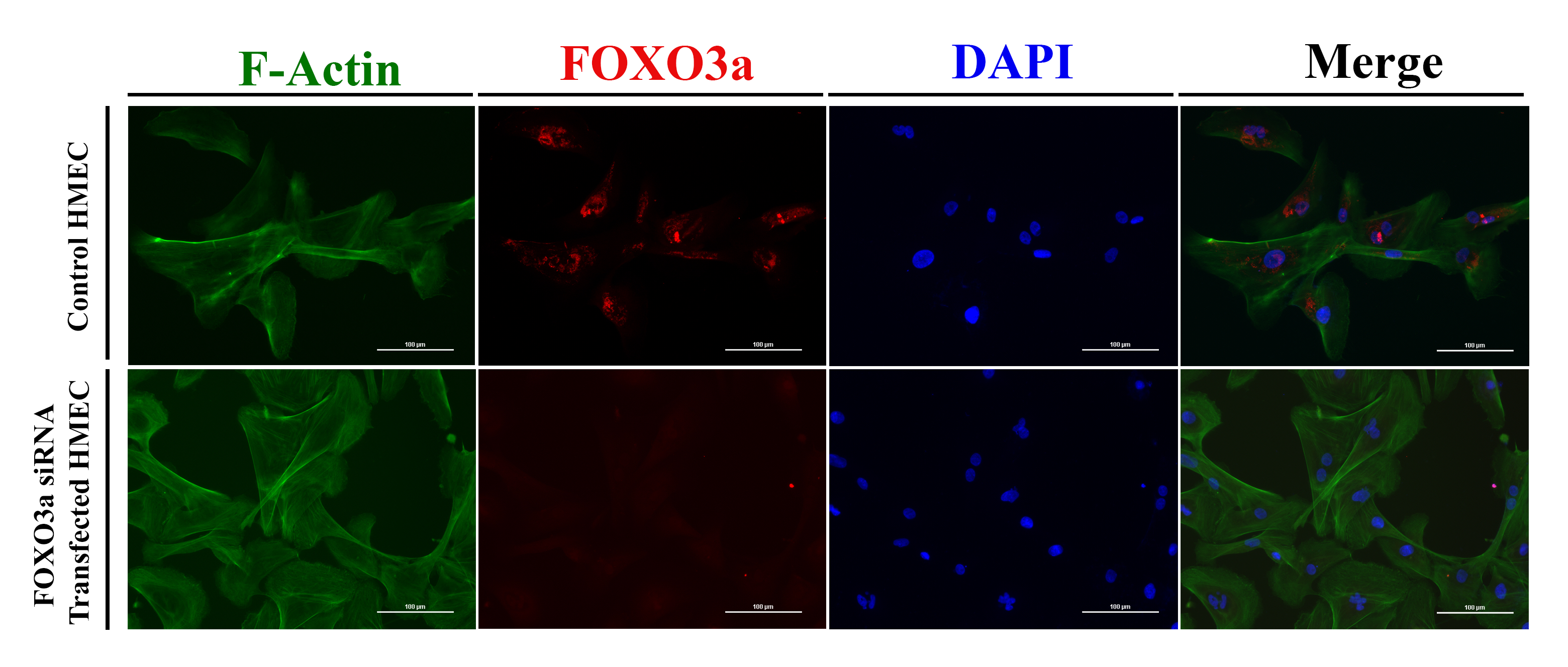
**

**Figure S2-Related to Figure 5: RNAi-mediated silencing of FOXO3a in HMEC cells.** FOXO3a expression was analyzed using immunofluorescence assay.


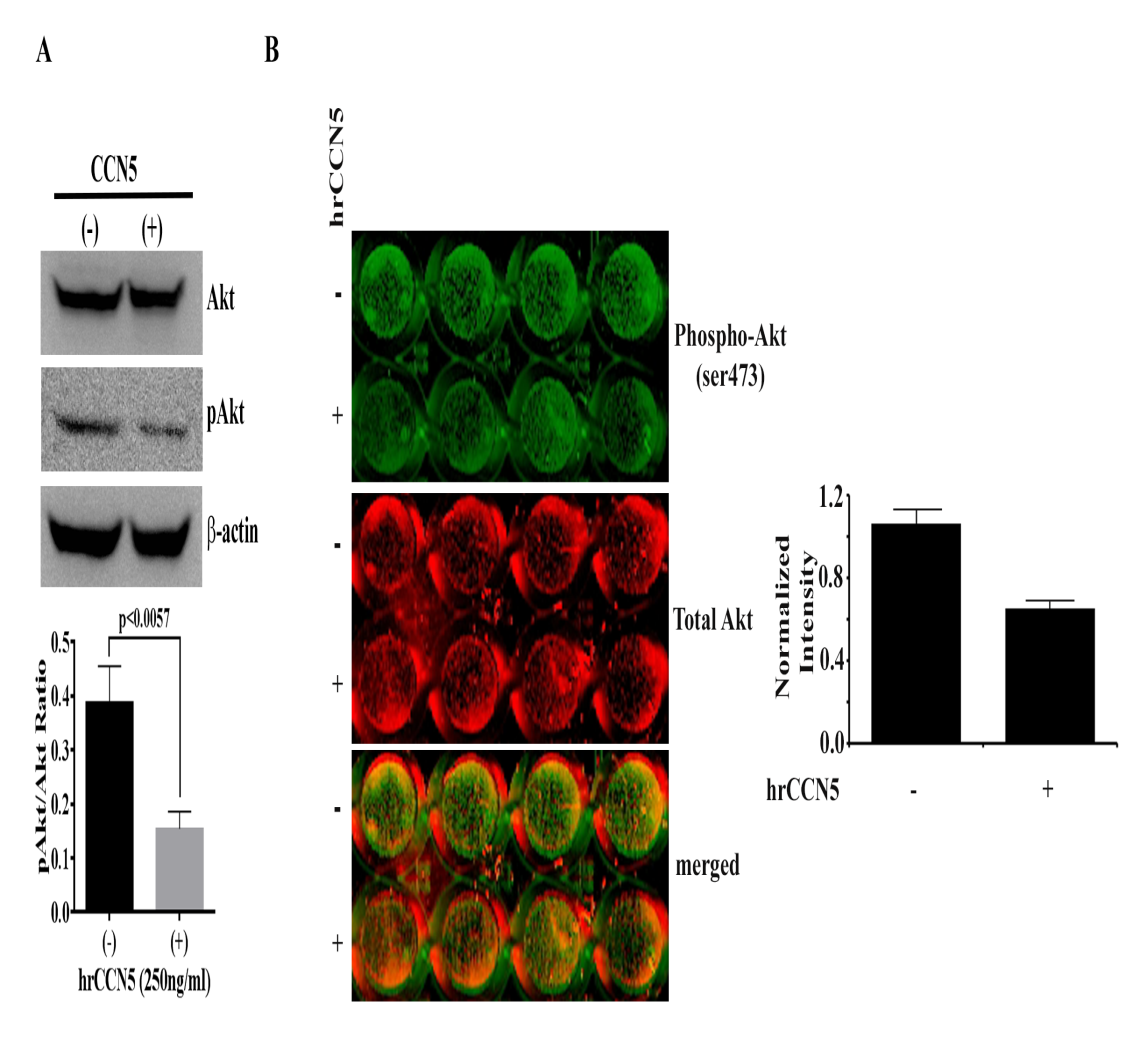


**Figure S2- Related to Figure 5: Quantitative analysis of Akt and p-Akt in hrCCN5-treated MDA-MB-231 cells. (A)** Western blot analysis and **(B)** In-cell Western blot analysis with Relative labeling intensity in different experimental samples. All data represent means ±SEM of three or 4 independent experiments. P values were calculated using one way ANOVA and two-tailed unpaired Student’s t-test.

Methods

*In-Cell Western blots:* In-cell Western blot assay was performed according to the protocol published by Whittaker et al. (Mol Cancer Ther; 14(12); 2700–11.). Briefly, about 10x10^4^ MDA-MB-231 cells were seeded into 96-well plates. After 24 h, cells were treated with hrCCN5 or left for 48 h. Cells were then fixed and permeabilized with 4% formaldehyde, 0.1% Triton X-100 for 30 minutes. Wells were blocked with blocking buffer (1X PBS/5% normal goat serum/0.3% Triton X-100) for 60 min and then incubated with phosphoPlus AKT (Ser473) in-cell duet antibody (Cell signaling) overnight at 4^o^C. Cells were then washed with 0.1% Tween-20 and incubated with detection cocktail conjugated to IR dye for 1-2 h at room temperature in the dark. Cells were then washed three times with PBS for 5 min each. Finally, Plates were then read using an Odyssey scanner (LI-COR). Phospho-AKT was normalized to total AKT.

**Figure S4- Related to Figure 7: CCN5 ablation by shRNA in MCF-7 cells promotes desensitization to Tamoxifen.** To corroborate the role of CCN5 in catastrophic effect of Tam, semiconfluent MCF-7 cells were transiently transfected with CCN5-shRNA or scrambled-shRNA using Neon Transfection System (Invitrogen) as per manufacturer’s instruction and as described in the Materials and Methods section. Transfected cells were re-seeded in 96-well-plates. ~60% confluent cells were treated with Tam (10μM) for 48h and cell viability was measured. All data represent means ±SEM of eight independent experiments. P values were calculated using one way ANOVA and two-tailed unpaired Student’s t-test.

**Figure S5- Related to Figure 7: Body weight analysis of treated and untreated athymic nude mice with same age (~12 weeks).** The body weight was measured using Studylog^R^ softwares.

**Table S1:** Inducibility of CCN5 (transgene) expression in different organs was determined immunohistochemically following Dox (2mg/ml) treatment (3 times/week) as described under Materials and Methods section.

| Transgenic (Mouse) | **Breast**  Basal Inducible | **Liver**  Basal Inducible | **Lungs**  Basal Inducible | **Kidney**  Basal Inducible |
| --- | --- | --- | --- | --- |
| CCN5/M1  CCN5/M2  CCN5/M3  CCN5/M4  CCN5/M4 | +/- +++++  - ++++  +/- +++++  - +++++  - ++++++ | - -  - -  - -  - -  - - | +/- -  +/- -  - -  - -  +/- +/- | - -  - -  - -  - -  - - |

+, 1x positive, -, negative
